# Oxidative Stress: What Is It? Can It Be Measured? Where Is It Located? Can It Be Good or Bad? Can It Be Prevented? Can It Be Cured?

**DOI:** 10.3390/antiox11081431

**Published:** 2022-07-23

**Authors:** Angelo Azzi

**Affiliations:** School of Graduate Biomedical Pharmacology and Drug Development Program, Tufts University, Boston, MA 02111, USA; angelo.azzi@tufts.edu

**Keywords:** oxidative stress, oxidative damage, reactive oxygen species, antioxidants, cell signaling, eustress, distress

## Abstract

The meaning, the appropriate usage and the misusage of the terms oxidative stress, oxidative eustress, and oxidative distress have been evaluated. It has been realized that the terms oxidative stress and oxidative damage are often used inappropriately as synonyms. The usage of the term eustress (intended as good stress) is unsuitable to indicate signaling by reactive molecular an event that can be finalistically considered either good or bad, depending on the circumstances. The so defined oxidative distress is an oxidative damage but not an oxidative stress. What is measured and defined as oxidative stress is in fact an oxidative damage. Damaging oxidations and signaling oxidant events (good or bad) can be present, also simultaneously, in different and multiple location of a cell, tissue or body and the measure of an oxidant event in body fluids or tissue specimen can only be the sum of non-separatable events, sometimes of opposite sign. There is no officially approved therapy to prevent or cure oxidative stress or oxidative damage.

## 1. Oxidative Stress: What Is It?

**Figure 1:**
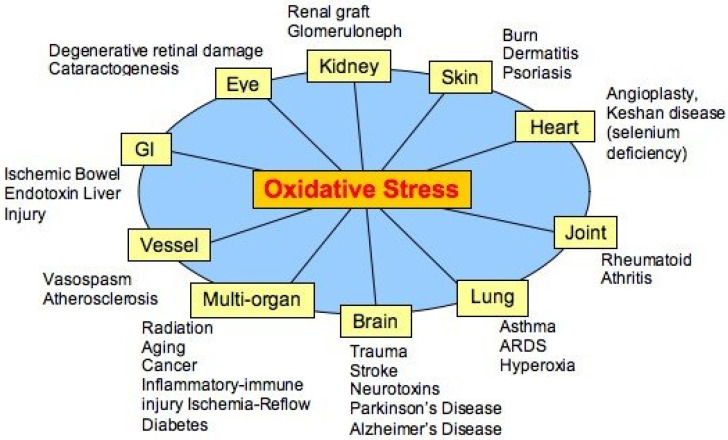
Alleged diseases linked to oxidative stress (modified from [6]).

The two definitions of “stress” from the Oxford Language Dictionary are: “pressure or tension exerted on a material object” and “a state of mental or emotional strain or tension resulting from adverse or demanding circumstances”. In medicine, Hans Selye was the first scientist to define ‘stress’ as being at the bottom of generic signs and symptoms of illness (1956). “Stress”, combined with “oxidative”, was first used in oxidative stress relaxation of a material [1] and was related to the elasticity of rubber. The term “oxidative stress” was first mentioned in the biological literature in 1970 [2]. The concept of biological oxidative stress was developed in more detail later in “Oxidative stress: a concept in redox biology and medicine” [3], and it was defined as “an imbalance between oxidants and antioxidants in favor of the oxidants, leading to a disruption of redox signaling and control and/or molecular damage” [4]. At that time, when free radicals as having a possible role in, and were considered primary agents of many diseases, (Figure 1) oxidative stress was a much-welcomed concept linking the damaging role of free radicals with the protective role of endogenous and exogenous antioxidants.

Since then, a surprising number of articles on “oxidative stress” have been published. In the database PubMed, 281,726 articles using this term were found (the search was conducted on 6 June 2022). It is surprising that the popularity of the term surpassed that of mitochondria (239,148), of Lysosome (91,989), of Golgi (53,003) or Double Helix (8279) and was just a bit less than vitamin (447,650). Searching for the combination of “oxidative stress and disease”, 111,807 articles were found, only 56% of those with the keyword Alzheimer’s (198,497), and 40% of those with the keyword “Heart Infarction” (279,182).

Such an extraordinary success of the term “oxidative stress” has prompted an in-depth analysis of this phenomenon, the conclusions of which are reported in the following paragraphs.

As oxidative stress is a term used in biological science, the first question that can be asked is which biological components can be affected by such an imbalance between production of radicals (or more generally, oxidants and reactive oxygen species) and their elimination. The answer is simple: reactive oxygen species can originate in all organelles, cells and tissues, and all, from molecules to organelles and tissues and organisms, can be affected. The next question is whether this imbalance between oxidants and antioxidants in favor of the oxidants can be measured.

## 2. Oxidative Stress: Can It Be Measured?

Oxidative stress is a historical concept from the time when all diseases were considered as being caused by too much free radical production or too little elimination; it was not supposed to be measured but “cured” by antioxidants. However, large-scale intervention trials using antioxidant supplements provided with free radical scavenging capacity have shown no significant advantage in humans. Consequently, a new hypothesis for oxidative stress in disease had to be formulated: Oxidative stress occurs due to alteration of thiol redox circuits, which normally play a role in cellular signaling and physiological regulation [5]. However, both with the definition of oxidative stress as an imbalance between oxidants and antioxidants in favor of the antioxidants [3] and the concept of redox disruption by oxidants, the measurement of the rates of oxidants’ production vs. that of oxidants’ elimination appears to be necessary. In principle, these measurements are possible, and a number of methods have been developed towards this goal, in the hope of understanding the role of oxidative stress in human disease. Measurement of disease-associated oxidative stress is important, although it would not solve the question whether oxidative stress is a cause or consequence of disease [6].

A number of techniques can be used (available online: https://www.cellbiolabs.com/oxidative-cellular-stress (accessed on 17 June 2022) depending on the sample type (cells, tissues, blood, urine, food samples). Most of the tests measure the damage that radicals or excess oxidants have caused, such as DNA/RNA damage (8-Hydroxyguanosine (8-OHG), 8-Hydroxydeoxyguanosine (8-OHdG), Abasic (AP) sites, BPDE DNA Adduct, Double-strand DNA breaks, and Comet Assay (general DNA damage)). Other techniques analyze the product of lipid damage as a result of radical or oxidant impact (lipid peroxidation 4-hydroxynonenal, 8-isoprostane, malondialdehyde (MDA), and TBARS). Additionally, protein modifications have been used, such as oxidation/nitration, protein carbonyl content (PCC) 3-nitrotyrosine, advanced glycation end products (AGE), advanced oxidation protein products (AOPP), protein adducts, methylglyoxal adducts, protein radicals, and s-glutathione adducts.

All these techniques proposed to measure oxidative stress are really measuring the damage produced by an oxidative event and not the rate of damage versus the rate of protection.(Table 1) Essentially the above static techniques do not measure oxidative stress, which is a dynamic concept. Other techniques have also been proposed to measure the molecules that cause damage: reactive oxygen species (ROS), reactive nitrogen species (RNS), hydrogen peroxide, and nitric oxide. These measurements have the drawback that the molecules in question are subject to rapid and uncontrolled decay. Attempts are made to measure the antioxidants function of enzymes such as catalase, glutathione, superoxide dismutase or of complex mixtures (Oxygen Radical Antioxidant Capacity (ORAC), Hydroxyl Radical Antioxidant Capacity (HORAC), Total Antioxidant Capacity (TAC). All these techniques have been strongly criticized. The concept of “total antioxidant capacity (TAC), which originated from chemistry and then was applied to biology and medicine, and further to nutrition and epidemiology, needs critical appraisal, because there are serious limitations that preclude meaningful application to in vivo conditions” [7].

Some of the tests (antioxidant capacity assays), which use, for instance, the iron or copper reduction capacity of a given sample to measure the antioxidant power (FRAP), hydroxyl radical antioxidant capacity (HORAC), oxygen radical antioxi-dant capacity (ORAC), Trolox equivalent antioxidant capacity (TEAC), and total antioxidant capacity (TAC), have been strongly criticized. Most of the assays do not measure the imbalance between oxidants and reductants according to the definition of stress, but they measure the damage produced by either free radicals or oxidants. The modification of nucleic acids, proteins, lipids, sugars and low molecular weight substances is in fact a damage, and calling it oxidative stress is improper. What is clear from all the above considerations? Oxidative stress cannot be measured, being an imbalance; an imbalance in fact may cause damage, but has not yet caused damage, or may never cause damage.

## 3. Oxidative Stress: Where Is It Located?

Oxidative damage, as measured in a biological sample (urine, serum, liquor) but also in a tissue sample, can be the sum of different oxidative events, in different parts of a cell, in different tissues or organs. From the global measurements of oxidative damage in a biological sample, one cannot infer if it occurred at a single site or at multiple and different ones. In addition, the oxidative event may result in consequences that depend on the location of the damage (molecular damage, cell damage or up- and downregulation of signaling pathways). Due to this uncertainty, it is questionable if the measurements of oxidative damage in vivo can have a diagnostic and/or prognostic value. A total measure of oxidant modification in a simple or complex organism is therefore not useful [8]. However, the question can be asked as to what information can be obtained by measuring oxidative stress in cells in vitro. 

The traditional way to induce an oxidative stress in cells in vitro is that of adding an oxidant, frequently hydrogen peroxide, to a cell culture. By this approach, cells receive an overall strong oxidative burst, far from what happens in a pathophysiological setup. Although frequently used, such a method has been the object of serious critiques [9].

**Figure 2:**
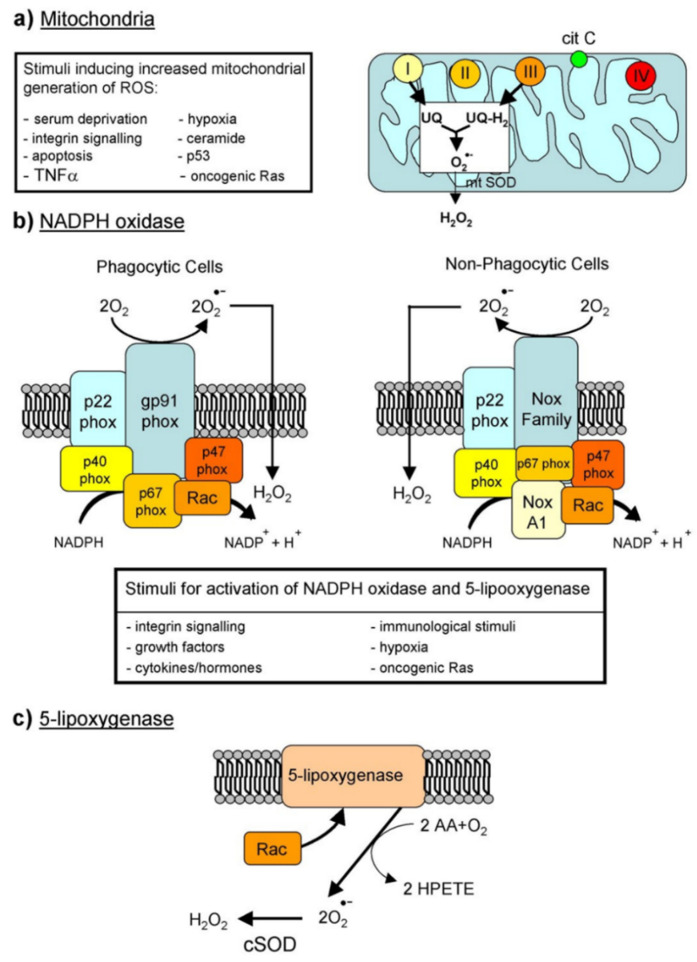
Cellular production of reactive oxygen species Reproduced from [10].

Under physiological conditions (Figure 2), mitochondria, cell membrane-associated NADPH-oxidases and lipoxygenases produce either superoxide (then dismutated to hydrogen peroxide) or hydrogen peroxide directly. Formation of highly reactive hydroxyl radicals from hydrogen peroxide in the presence of free iron or copper is possible. Hydrogen peroxide at variance with highly reactive oxygen radicals can also initiate a signaling pipeline of redox networks.

Superoxide is too self-reactive to diffuse to any major distance, but can be released to the exterior of the cell. It is very unstable and rapidly dismutates to produce peroxide, which is then protonated to become hydrogen peroxide. Hydrogen peroxide can diffuse to longer distances with respect to superoxide, although limited by adjacent peroxidases, it can be secreted and imported into cells through aquaporin (AQP). Hydrogen peroxide oxidizes thiol residues in protein tyrosine phosphatases. The resulting inhibition increases tyrosine phosphorylation on target proteins. Oxidative burst is the pathophysiological production of hydrogen peroxide in phagocytic cells as antimicrobial innate immune response; its production in phagosomes has the important role of killing phagocyted bacteria and fungi. Gastrointestinal low hydrogen peroxide concentrations trigger anti-virulence mechanisms; in the thyroid, hydrogen peroxide is used to oxidize iodide and synthetize the active thyroid hormone.

A significant production of reactive oxygen species is due to the activity of the NADPH oxidases (nicotinamide adenine dinucleotide phosphate oxidase). They are enzyme complexes associated with membranes pointing to the extracellular space. Both the plasma membrane and the phagosomes host some isoforms of NADPH oxidases (in humans, NOX1, NOX2, NOX3, NOX4, NOX5, DUOX1, and DUOX2). The stimulated NADPH oxidases produce superoxide, which takes part in animal immune response and cell signaling.

NADPH oxidase activity is precisely regulated to keep a physiological concentration of reactive oxygen species in the body. The NADPH oxidase family members are inactive in resting cells but become quickly activated by several stimuli, including bacterial products and cytokines. Vascular NADPH oxidases are regulated by a variety of hormones and factors, important in vascular remodeling and disease. They comprise thrombin, platelet-derived growth factor (PDGF), tumor necrosis factor (TNFa), lactosyl-ceramide, interleukin-1, and oxidized LDL. The activity of NADPH oxidase in the vascular system is increased by agonists and arachidonic acid. Instead, enzyme assembly is prevented by apocynin and diphenylene iodonium. Other sources of reactive oxygen species include the activity of cytochromes P450, xanthine oxidase, peroxisomal oxidases, and lysyl oxidase. From what has been described above, the multiple localization of the generators of oxidative events in the body makes total body measurement not meaningful for understanding potential underlying pathologies.

## 4. Oxidative Stress: Can It Be good or Bad?

With the emerged knowledge that reactive oxygen species can be employed in fine cell regulation the term oxidative stress has been subdivided in “oxidative eustress”, providing beneficial signaling and “oxidative distress” capable of producing chemical damage [11].

Since redox signaling and redox regulation can be modulated by physiological (low-level) amounts of reactive oxygen species, this has been defined as “oxidative eustress”, whereas higher loads (supraphysiological) of oxidant species bring about disrupted redox signaling and/or oxidative damage to biomolecules; the latter has been defined as “oxidative distress” [11]. The name “eustress” is derived from the ancient Greek “
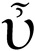
ὖ“, which means good, and it is opposed by the negative “distress”, from the Latin “dis-“ meaning apart or away. Lower amounts of oxidants can play a role in regulating several biochemical reactions, for example, hydroxylating, carboxylating, or peroxidating reactions, or in the reduction of ribonucleotides. They also have important modulating and regulatory functions in the signal transduction of intercellular information [12,13].

The complex and fine regulation of cellular production of reactive oxygen species is therefore physiologically essential, but no stress is associated with such reactions. Studies have shown that low levels of reactive oxygen species can be associated with redox signaling, with a positive role in physiology and health [14]. However, low levels of reactive oxygen species can promote cancer survival [15,16]. The production of reactive oxygen species at higher intensity can indeed produce damage to biomolecules with potentially deleterious outcome and disease, “oxidative distress”, but can also lead to a positive outcome with the apoptotic death of cancer cells [17]. Large amounts of reactive oxygen species play a finalistically seen positive role in phagocytosis due to the microbicidal function of this systems. Local production of reactive oxygen species at higher intensity is also physiologically responsible for T4 synthesis in the thyroid. The inhibition of these higher intensity oxidative events implied in physiological effects may result in disease.

In conclusion, the separation of low oxidative events in a physiologically positive actions and the high oxidative events in negative actions cannot have a general application. Oxidative eustress and oxidative distress are not chemical situations, but global, abstract concepts that cannot be chemically distinguished, in light of the fact that the actors are the same in all cases, i.e., oxidative species. However, reactive oxygen species can have a regulatory function (finalistically seen as good or bad) or can have a damaging function (also, in this case, finalistically seen as bad or good). Preconditioning is a mechanism by which small amounts of an oxidant provided to cells at different times and in different amounts result in a resistance to the damage provided by high amounts of oxidants. This is an example of the difficulty in defining high and low oxidant amounts and their relative function [18,19].

All those events, called stress, may be topographically different, the damage or the regulation (good or bad) being exclusive to a specific place and occurring at the same time in different and multiple locations.

## 5. Oxidative Stress: Can It Be Prevented?

Protection against membrane and lipoprotein–lipid peroxidation should in principle be afforded by antioxidants. However, the information obtained in vitro on an antioxidant cannot be extended to cells, to organs, to animals or to populations until proof is provided. A molecule that has been shown to have antioxidant properties in vitro may have alternative, opposite (pro-oxidant), or additional properties in an integrated system. A few examples are given in the next paragraphs. In vitro studies have suggested that estrogens prevent lipoprotein oxidation and provide antioxidant neural protection. Instead, receptor-mediated signaling is the mode estrogens act in vivo and not by exerting weak antioxidant effects. Although retinol exhibits antioxidant properties in vitro, its in vivo function is related to its binding to opsin, which forms rhodopsin, the pigment required for vision. It has been claimed that melatonin has antioxidant functions, but the concentrations available in the human body (sub-micromolar) cannot have antioxidant effects. Genistein is a soy iso-flavonoid; it inhibits protein-tyrosine kinase and topoisomerase-II (DNA topoisomerase (ATP-hydrolyzing)), and induces G2 phase cell arrest but not as an antioxidant. Curcumin anti-inflammatory properties have been shown in some in vivo studies. However, after O-conjugation, its antioxidant property remains but not its anti-inflammatory function, indicating that the anti-inflammatory property of the molecule is dissociated from its antioxidant function. Another problem associated with natural antioxidants uptake in the diet is their low bioavailability relative to the amount of cell-generated oxidants; in addition, the small amounts present in the body have not been shown to be regenerated after their oxidation by reactive oxygen species. Tea polyphenols EGCG, EGC, and (2)-epicatechin (EC), are probably responsible for the beneficial effects of tea. However, they are not freely available to exert an antioxidant effect since almost all catechins in plasma are highly conjugated. In addition, just introducing an antioxidant molecule into the body does not imply that it is active as antioxidant: on uptake of flavonoid-rich foods, a plasma antioxidant burst is observed; however, it is not caused by the flavonoids, but by concomitant increase in uric acid [20].

As indicated in the previous paragraphs, several reasons have been brought to support the idea that it is questionable whether flavonoids and their metabolites can function as major antioxidants in vivo. However, sufficiently high concentrations of flavonoids can be reached in vivo to allow them to display pharmacological activity at receptors, enzymes, and transcription factors. It should also be underlined that the amount and velocity of production of reactive oxygen species in a physiological context is exactly controlled and possess specific targets such as redox-dependent factors, creating innumerable pathways in which oxidants act as effectors in various processes, from tumorigenesis to ageing. Superfluous or uncontrolled administration of antioxidants may result in the malfunctioning of cellular pathways implicated in vital signaling [21].

The antioxidant properties of vitamin E have been well documented in vitro and vitamin E has for a long time been considered one of the most important physiologically active antioxidants, needed to prevent all diseases allegedly based on oxidative stress. At the basis of the antioxidant properties of vitamin E is its ability to quench fatty acid peroxyl radicals in membranes and lipoproteins, where the hydrophobic molecule dissolves, by becoming an α-tocopheroxyl radical itself. The potential damage by α-toco-peroxyl radicals and the rapid consumption of the vitamin would be avoided by α-toco-peroxyl radicals’ reduction by a suitable reducing agent. Thus, the combination of vitamin E, absorbed from nutrients in small amounts and ascorbic acid (or lipoic acid), would protect membranes from losing their structure and protect oxidized lipoproteins from becoming atherogenic. Such a mechanism has been demonstrated in numerous in vitro systems. Experiments in cell cultures, in erythrocytes, in hepatocytes, in lipid bilayers, in micelles, and in low-density lipoproteins have shown that ascorbate saves cellular α-tocopherol through its recycling. However, supplementation in humans has provided less-clear results. Based on the assumption that increased lipid peroxidation is associated with accelerated atherogenesis, supplementation with the combination of vitamin E and ascorbate should provide protection. In a study [22], supplementation of individuals susceptible to accelerated atherosclerosis with α-tocopherol resulted in the near doubling of its plasma concentrations, but additional supplementation with ascorbic acid did not significantly increase the basal level of vitamin E. This suggests that there is no in vivo consumption of vitamin E and ascorbic acid is not providing a recycling/rescuing effect. The National Academies, USA [23], have stated that “the extent to which vitamin E is recycled in humans and which antioxidant species are preferentially used for recycling is not known”. A-Tocopherol quinone in humans is produced as an oxidation derivative of vitamin E; its presence has been taken as evidence that vitamin E protects as antioxidant against the oxidation of biological molecules induced by reactive oxidation species [24]. This study has shown that vitamin E can be oxidized in vivo, but vitamin E oxidation does not result in protection against supposed lipid, protein or DNA damage-associated diseases. Absurdly, it can be contended that DNA can also be oxidized, its oxidation products measured, and the oxidative damage repaired [25], but this does not imply that DNA is an antioxidant.

It has been shown that high-dose RRR-α-tocopherol administered to patients with coronary artery disease significantly reduced plasma biomarkers of oxidative damage but had no significant effect on carotid intima-media thickness during the 2 years of treatment [26]. Essentially, the measured oxidative damage appeared to be dissociated from the pathological situation since it appears that vitamin E, as an antioxidant, is not able to protect against diseases that are allegedly based on oxidative stress; this brings us to the conclusion that either vitamin E is not acting against oxidative stress or that the diseases are not caused by oxidative stress. In more general terms, no antioxidant has been shown to protect against whatever disease, and no medical doctor is obliged to prescribe an antioxidant for whatever medical situation. As an additional consideration, excess antioxidants can be responsible for health negative effects [27].

For almost a century, the expectations of antioxidants as health-supporting mediators were very high. However, meta-analyses of clinical studies have shown that supplementation of antioxidants does not result in the expected health benefit, but is connected in some cases with increased mortality [28]. In 68 randomized trials, the effects of β-carotene, vitamin A and vitamin E on mortality had no beneficial effects. These supplements even augmented all-cause mortality and, in particular, excess carotenoids and vitamin E have been associated with cancer growth and other health impairments [28]. As to the protection against kidney disease, at present, there is insufficient evidence to recommend increasing intake of vitamin E in patients with chronic kidney disease [29].

There is building evidence that antioxidant supplementation can attenuate endurance training. In fact, reactive oxygen species mediated mitochondrial biogenesis, cellular defense mechanisms and insulin sensitivity [30] would be blocked by antioxidants. 

The effect of oxidative stress on different pathological situations can be summarized as follows. Although signs of oxidative damage have been found to be associated with some diseases [31] a cause-to-effect relationship has not been demonstrated in clinical setups. Randomized clinical trials found that vitamin E supplements provided no differences in rates of overall cardiovascular events. Human trials and surveys have found that vitamin E is not beneficial in most cancers. Vitamin E supplements, taken alone or in combination with other antioxidants, cannot reduce the risk of developing AMD or cataracts. Most studies do not support the use of vitamin E supplements by healthy or mildly impaired individuals to maintain cognitive performance or slow its decline with normal aging. In addition, high doses of alpha-tocopherol supplements can cause hemorrhage and increase the risk of prostate cancer [32].

## 6. Oxidative Stress: Can It Be Cured?

After having discussed the evanescent concept of oxidative stress and that more concrete concept of oxidative damage, which can be measured in some pathological situations, the appropriate question should be: Can oxidative damage be repaired?

Pharmacological use of antioxidants is being considered, and some clinical trials are presently under way. These comprise the following: elimination of O_2_^•−^ before its reaction with ^•^NO to form ONOO^-^ and removal of hydrogen peroxide before it reacts to give ^•^OH; increasing GSH, using precursors; stimulating the synthesis of antioxidant enzymes, particularly through NRF2 activation; inhibition of NOXs; increasing mitochondrial antioxidant defense; supplementing dietary antioxidants; finally, inhibiting aberrant redox signaling (Forman and Zhang, 2021). Until now, no clinical use of any of these approaches is recommended or even considered.

In conclusion, to the questions posed in the title—What is it? Can we measure it? Can it be good or bad? Where is it located? Can it be prevented? Can it be cured?—the following answers can be given.

What is it? Although the theoretical definition is clear, namely, “an imbalance between oxidants and antioxidants in favor of the oxidants”, practically speaking, there are a number of issues related to the good and bad oxidative stress: in fact “good” eu-stress may cause negative consequences and “bad” stress or distress is not an imbalance but just an oxidative damage. In addition, oxidative distress can also be finalistically seen as good or bad.

Can we measure it? The measure of the stress is in fact a measure of damage and not of an imbalance that could cause damage. The subtle physiological changes in the local levels of reactive oxygen species cannot be measured, being highly localized and possibly with diverging signaling properties. The question can be posed whether an increase in reactive oxygen species in a cell is good or bad for the body; the answer can be good, as an increase in reactive oxygen species results in increased bacterial defenses, or bad if reactive oxygen species signaling will produce unwanted effects, for instance, cancer growth. Good or bad oxidative stress and wanted or unwanted effects cannot be separately measured. It is clear that a sustained high level of reactive oxygen species produced in whatever form in the body can only be a detrimental element for health.

Can it be prevented? Can it be cured? Originally, the idea has been that soluble antioxidants (vitamins, biofactors) could directly scavenge the reactive oxygen species and prevent their negative consequences. In fact, no data have shown that the diseases, allegedly caused by oxidative stress, can be prevented by vitamins or antioxidant biofactors. It may be argued that those diseases are not caused by oxidative stress. However, since the list of diseases at the basis of which an oxidative stress is assumed comprises practically all diseases, the conclusion would be that no diseases are caused by oxidative stress. It has been argued that the so-called soluble antioxidants would not be absorbed and reach their targets in sufficient amounts. By increasing the doses of soluble antioxidants such as vitamin E or beta-carotene, negative consequences (increase in disease instead of protection) have been obtained. Another aspect to be considered is that adding massive amounts of antioxidants to the body may result in neutralizing positive signals produced by reactive oxygen species. Considering that the path to soluble antioxidants is virtually unpracticable, the paradigm has been revisited by stating that exogenous, soluble antioxidants are in fact not useful, as the endogenous antioxidant system is of much greater value. The system that has been described as a first line antioxidant defense basically includes superoxide dismutase (SOD), catalase (CAT) and glutathione peroxidase (GPX). Without doubt, these enzymes are intended to comprehensively eliminate a source of damage, hydrogen peroxide, but their activity must be locally dosed to avoid the signally properties of hydrogen peroxide being suppressed by its excessive catabolism. The goal of manipulating the sophisticated network of cellular oxidant and antioxidant enzymes, by increasing or diminishing their activity, appears to be bound with enormous risks.

Finally, it appears that the concept of oxidative stress, with its variations in eustress and distress, represents an interesting pathophysiological hypothesis, albeit with a number of caveats. However, the use of the oxidative stress notion in complex systems, and even more so in clinical studies, can only be considered a fig leaf used to cover the unknown mechanisms of disease pathogenesis.

## Figures and Tables

**Table 1:** The different antioxidant assays.

Antioxidant Capacity Assays
Antioxidant Enzyme Activity Assays
Ascorbic Acid Assay (FRASC)
Cell Based Exogenous Antioxidant Assay
Chitosan Assay Kit
Flavonoid Assay
Glutathione Assays
Hydrogen Sulfide Gas Assay
Total Thiol Assay
DNA/RNA Damage and Repair
8-OHG RNA Damage ELISA
8-OHdG DNA Damage ELISA
AP Sites Quantitation Kit
Comet Assays and Slides
DNA Double-Strand Break Assay
Poly (ADP-Ribose) ELISA
UV-Induced DNA Damage Assays
Lipid Peroxidation
8-iso-Prostaglandin F2a Assay
HNE (4-Hydroxynonenal) Assays and Reagents
MDA (Malondialdehyde Assays and Reagents
Oxidized HDL ELISA Kits
Oxidized LDL ELISA Kits
Oxidase/Peroxidase Activity Assays
Monoamine Oxidase Assays
Myeloperoxidase Activity Assays
Peroxidase/Hydrogen Peroxide Assays
Polyamine Oxidase Assay
Protein Oxidation/Nitration
Advanced Glycation End Products
Advanced Oxidation Protein Products (AOPP) Assay
Oxidized/Modified Human Lipoproteins
Protein Carbamylation Assays and Reagents
Protein Carbonyl Assays and Reagents
Protein Nitration Assays and Reagents
Protein Radical ELISA
S-Glutathione Protein Adduct ELISA
Reactive Oxygen Species (ROS) Assays
Hydrogen Peroxide Assays
Nitric Oxide Assays
Universal ROS and RNS Assays
